# Immersive Technologies for Depression Care: Scoping Review

**DOI:** 10.2196/56056

**Published:** 2024-04-25

**Authors:** C Mahony Reategui-Rivera, David Villarreal-Zegarra, Kelly De La Cruz-Torralva, Paquita Díaz-Sánchez, Joseph Finkelstein

**Affiliations:** 1 Department of Biomedical Informatics University of Utah Salt Lake City, UT United States; 2 Instituto Peruano de Orientación Psicólogica Lima Peru; 3 Escuela de Psicología Universidad Continental Lima Peru; 4 Unidad de Telesalud Facultad de Medicina Universidad Nacional Mayor de San Marcos Lima Peru

**Keywords:** depression, immersive technologies, virtual reality, augmented reality, mobile phone

## Abstract

**Background:**

Depression significantly impacts quality of life, affecting approximately 280 million people worldwide. However, only 16.5% of those affected receive treatment, indicating a substantial treatment gap. Immersive technologies (IMTs) such as virtual reality (VR) and augmented reality offer new avenues for treating depression by creating immersive environments for therapeutic interventions. Despite their potential, significant gaps exist in the current evidence regarding the design, implementation, and use of IMTs for depression care.

**Objective:**

We aim to map the available evidence on IMT interventions targeting depression treatment.

**Methods:**

This scoping review followed a methodological framework, and we systematically searched databases for studies on IMTs and depression. The focus was on randomized clinical trials involving adults and using IMTs. The selection and charting process involved multiple reviewers to minimize bias.

**Results:**

The search identified 16 peer-reviewed articles, predominantly from Europe (n=10, 63%), with a notable emphasis on Poland (n=9, 56%), which contributed to more than half of the articles. Most of the studies (9/16, 56%) were conducted between 2020 and 2021. Regarding participant demographics, of the 16 articles, 5 (31%) exclusively involved female participants, and 7 (44%) featured participants whose mean or median age was >60 years. Regarding technical aspects, all studies focused on VR, with most using stand-alone VR headsets (14/16, 88%), and interventions typically ranging from 2 to 8 weeks, predominantly in hospital settings (11/16, 69%). Only 2 (13%) of the 16 studies mentioned using a specific VR design framework in planning their interventions. The most frequently used therapeutic approach was Ericksonian psychotherapy, used in 56% (9/16) of the studies. Notably, none of the articles reported using an implementation framework or identified barriers and enablers to implementation.

**Conclusions:**

This scoping review highlights the growing interest in using IMTs, particularly VR, for depression treatment but emphasizes the need for more inclusive and comprehensive research. Future studies should explore varied therapeutic approaches and cost-effectiveness as well as the inclusion of augmented reality to fully realize the potential of IMTs in mental health care.

## Introduction

### Background

Depression is a debilitating disorder characterized by a persistent low mood and a loss of interest in everyday activities, significantly affecting various dimensions of life [1]. Globally, approximately 280 million people are afflicted by this condition [2]. However, only 16.5% of people with depression worldwide receive treatment, indicating a substantial treatment gap [3]. The scarcity of mental health professionals exacerbates this issue, with figures in low- and middle-income countries being particularly low at 1.4 to 3.8 per 100,000 population [4]. This shortage of resources highlights the urgent need for innovative solutions in mental health care [5].

Digital technologies, now more crucial than ever, have emerged as vital tools in bridging health care gaps [6]. Among these, immersive technologies (IMTs) such as virtual reality (VR) and augmented reality (AR) stand out for their potential to revolutionize depression care. These technologies offer computer-generated immersive experiences that blend virtual and real environments, with VR providing entirely virtual experiences and AR overlaying virtual objects onto the real world [7].

The application of IMTs in mental health leverages their ability to create controlled immersive environments, offering a safe space for individuals to explore coping exercises and techniques [8,9]. This digital modality encompasses immersive sensory experiences that allow users to interact with a virtual environment [10,11]. Such interactions have been shown to increase engagement in health care–related tasks [12,13], which is a crucial challenge in the treatment of anxiety and depression [14], providing a novel approach to mental health care. Furthermore, continual improvements in IMT device technology, exemplified by the Meta Quest VR headsets, have further broadened the accessibility of these interventions globally. Moreover, IMTs have proven effective in treating a wide range of mental health conditions, such as anxiety, posttraumatic stress disorder, autism spectrum disorders, and various phobias [15-22].

### Objectives

Although some reviews have examined the use of IMTs in treating depression [23-25], they have not focused primarily on depression as the assessment goal; nor have they focused on IMT applications specifically aimed at treating depression or on the psychotherapeutic aspects of these interventions. Moreover, the available literature did not address relevant elements, such as the design or implementation of IMT interventions.

Therefore, we aim to map the most rigorous available evidence on IMT interventions targeting depression treatment and identify the gaps related to the design and implementation of these interventions. Given the emerging nature of IMTs in mental health and our specific research focus, a scoping review was deemed the most appropriate methodology. This approach allows for a broad overview of the existing literature, identifying key concepts and highlighting gaps in the research. Furthermore, we decided to focus on randomized clinical trials (RCTs) to ensure a robust and reliable evidence base. RCTs are considered the gold standard in clinical research, providing high-quality data to inform clinical practice and guide future research. By concentrating on RCTs, we aim to capture the most rigorous and scientifically valid studies, thereby enhancing the credibility and applicability of our findings in this emerging area of mental health care.

## Methods

### Overview

This scoping review adheres to the framework formulated by Arksey and O’Malley [26], expanded upon by Levac et al [27] and Daudt et al [28] and summarized by the JBI Manual for Evidence Synthesis [29]. Accordingly, we followed five main steps for conducting the scoping review: (1) identifying the research questions; (2) identifying relevant studies; (3) selecting the studies; (4) charting the data; and (5) collating, summarizing, and reporting the results.

Regarding reporting, our study aligns with the PRISMA-ScR (Preferred Reporting Items for Systematic Reviews and Meta-Analyses extension for Scoping Reviews) 2020 guidelines [30] (Multimedia Appendix 1). The protocol was preregistered on Open Science Framework [31].

### Step 1: Identifying the Research Questions

The primary research question guiding this study is *What is the available scientific evidence and what are the gaps that exist in this evidence concerning the use of IMTs to address depression among adults?*

The study also seeks to address the following secondary questions:

- From which regions or countries does the evidence come?
- Which technical aspects of IMTs have been reported in the evidence?
- What therapeutic approaches were used?
- What are the barriers and facilitators to implementing IMT interventions for depression treatment?
- What outcomes have been evaluated in studies examining the impact of IMT interventions on addressing depression?

### Step 2: Identifying Relevant Studies

We conducted a systematic search of the following electronic databases: MEDLINE (via PubMed), Scopus, Web of Science, Embase, PsycINFO (via EBSCO), IEEE Xplore, and Cochrane Library. The search strategy incorporated the keywords “virtual reality,” “augmented reality,” “depression,” and “randomized clinical trial.” An example of the search query performed in PubMed is presented in Textbox 1, and the search queries for each database are detailed in Multimedia Appendix 2. The search was limited to articles published in English and spanned from the inception of each database to October 10, 2023.

**PubMed search query.**

**Search query**

- (“Depression”[MeSH] OR “Depressive Disorder”[MeSH] OR depressive*[tiab] OR depression[tiab]) AND “Virtual Reality”[MeSH] OR “virtual reality”[tiab] OR “Augmented Reality”[MeSH] OR “augmented reality”[tiab] OR “VR headset”[tiab] OR “VR glasses”[tiab] OR “virtual environment”[tiab] OR “virtual world”[tiab] OR metaverse[tiab] OR meta-verse[tiab]) AND (“Randomized Controlled Trial”[Publication Type] OR “Randomized Controlled Trials as Topic”[MeSH] OR “randomized clinical trial”[tiab] OR RCT[tiab] OR (randomized[tiab] AND “clinical trial”[tiab]))

### Step 3: Study Selection

The inclusion and exclusion criteria are detailed in Textbox 2. Secondary studies were excluded, but their references were consulted to identify primary research studies that fulfilled our selection criteria. Similarly, protocols were not included, but registration IDs were consulted in the web to find preliminary or primary results published in articles.

**Inclusion and exclusion criteria (we defined immersive technologies as all augmented reality and virtual reality–only applications that belong to the degree of full immersion, according to the definitions provided in the literature [7,10]).**

**Inclusion criteria**

- Articles must include randomized clinical trials and be published in peer-reviewed journals.
- All participants must be aged at least 18 y.
- Depression must be a primary outcome measured either through clinical assessment or validated screening tests.
- At least 1 group of participants should have received, or should have been exposed to, immersive technologies using glasses, headsets, or other head-mounted display devices, with or without using other complementary devices.

**Exclusion criteria**

- Secondary studies (systematic, umbrella, narrative, and scoping reviews) and protocols were not considered.
- Articles in which the immersive technology interventions only focused on exercise as a treatment were not considered.

We searched the various databases of scientific articles and exported all records as RIS format files. These records were imported into EndNote X9 (Clarivate) for automatic and manual duplicate checking. Subsequently, the selection process was carried out on the Rayyan web platform (Rayyan Systems Inc) in 2 phases. First, the records were screened by title and abstract by independent reviewers using the platform. In the second phase, full-text evaluations were conducted to determine compliance with the inclusion criteria. Each document was assessed independently by a pair of reviewers (CMRR and KDC as well as CMRR and PDS) to ensure that they met the eligibility criteria. Review disagreements were solved through consensus, and a third reviewer (DVZ) made a final decision in case disagreements persisted. The reasons for exclusion were documented (Multimedia Appendix 3). Before the selection process, reviewers undertook a pilot test with 10 articles to standardize the process and gain expertise in using the Rayyan platform.

### Step 4: Charting the Data

Two pairs of reviewers (CMRR and KDC as well as CMRR and PDS) independently collected data using a collection form developed for the study protocol and refined at the data collection stage. The reviewers performed a pilot test with 2 documents to standardize information extraction criteria. The collected data included general and study characteristics (country of study, study design, participants’ characteristics, the type of depression outcome, and intervention and control descriptions), IMT intervention technical aspects (devices, the amount of time used and frequency of use, the setting of use, duration, IMT design framework consulted, and the degree of guidance), therapeutic approach used, and implementation characteristics (implementation framework used, implementation stage, and barriers and enablers). Study designs were categorized following the clinical trial classification formulated by Hopewell et al [32]. The implementation stages were defined as follows: (1) *preliminary*, if it was a pilot or feasibility study; (2) *implementation*, if it was mentioned that the RCT had been developed after a pilot or feasibility study; and (3) *unclear*, if there was no mention of it being a pilot or feasibility study, and there was no reference either to the results from these studies.

### Step 5: Collating, Summarizing, and Reporting the Results

We used a narrative approach to synthesize data [33]. We describe the information in the *Results* section using frequencies and percentages. Detailed information for each included article is presented in cross-tables. In addition, the geographic location of the studies is visualized as a bubble plot, categorized by year.

## Results

### Overview

**Figure 1:**
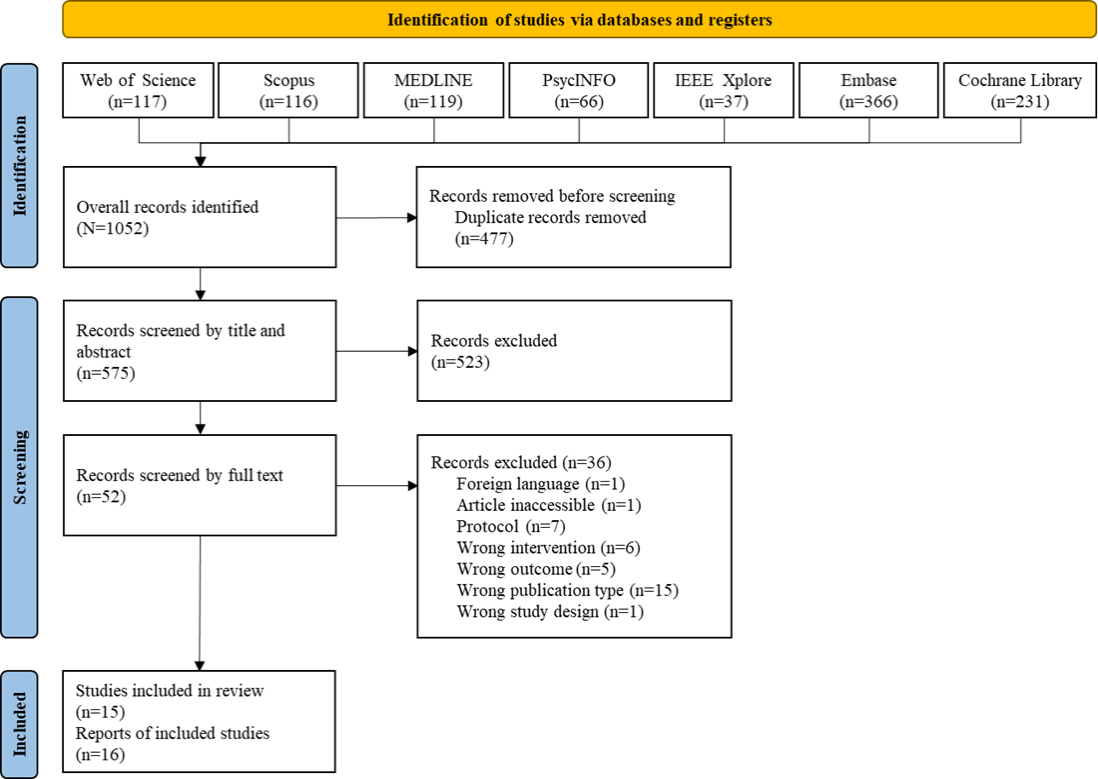
PRISMA (Preferred Reporting Items for Systematic Reviews and Meta-Analyses) 2020 flowchart outlining the search and selection process.

Our search strategy identified 1052 records; after removing 477 (45.34%) duplicates, the remaining 575 (54.66%) records were screened by title and abstract. Of these 575 records, 52 (9%) underwent full-text review. Of these 52 records, 36 (69%) were excluded, mainly on account of being the wrong type of publication (n=15, 42%); thus, 16 (31%) reports [34-49] from 15 studies were included in the review (Figure 1).

**Figure 2:**
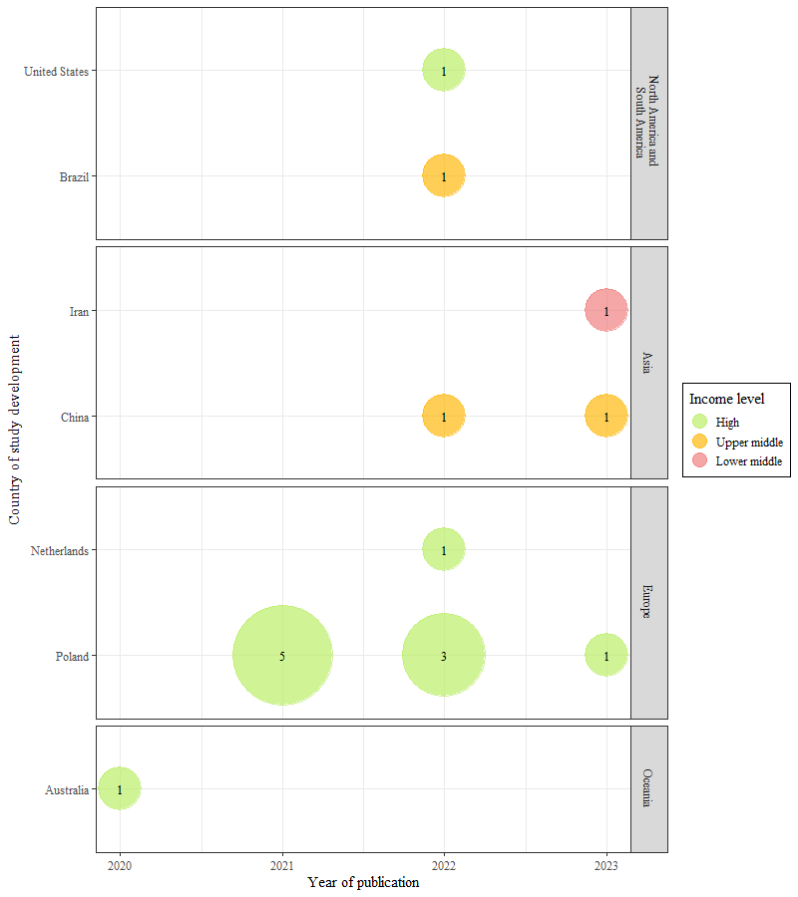
Trends of publication by geographic location of the reports.

Figure 2 illustrates an uneven distribution of research evidence on IMT interventions for depression management across different regions and income levels. European countries dominate the research landscape (10/16, 63%) [34,35,37-39,43-47], with Poland alone contributing more than half of the reports (9/16, 56%) [34,35,37-39,43-46] between 2021 and 2023. However, Poland showed a decreasing trend; of the 9 reports in this review, 5 (56%) were published in 2021, while only 1 (11%) was published in 2023. China maintains a steady presence in Asia with a study each in 2022 [48] and 2023 [49], amounting to 13% (2/16) of the total. By contrast, the United States [41], Australia [40], Brazil [42], and Iran (the only lower–middle-income country contributing to this research field) [36] contributed only 1 (6%) study each to the total of 16 studies. The trend analysis indicates a fluctuating global interest in the field, with a concentration of research in Europe (10/16, 63%) [34,35,37-39,43-47], intermittent contributions from other regions, and a spike in publications during 2022 (7/16, 44%) [35,39,41,42,44,47,48].

Table 1 illustrates the general characteristics of the selected reports. The majority (15/16, 94%) [34-40,42-49] adopted a parallel group design, while a single study (1/16, 6%) used a crossover trial design [40]. Among the 16 reports, only 1 (6%) featured 3 arms (2 control groups alongside 1 VR intervention group) [41]. Regarding the research timeline, most of the studies (9/16, 56%) were conducted between 2020 and 2021 [36,38,39,41-44,47,48]. However, for 3 (19%) of the 16 studies, the specific study period was not reported [35,37,45].

**Table 1:** Characteristics of included studies.

Reports	Year of study development	Country of study development	Study design	Participant characteristics	Specific depressive conditions studied and the measurement scale used	Sample size	VR^a^ group intervention description	Non-VR group intervention description	Effect
Cieślik et al [34], 2023	2022	Poland	Parallel group 2-arm randomized controlled trial+masked outcome assessor+2 time point measures (before and after the intervention)	Sex: female (60/60, 100%); age (y): mean 68.2 (SD 5.5)	Depression among older adults measured using the GDS-30^b^	60 (randomized; intervention: 30; control: 30)	General fitness training (40 min of low-intensity general fitness exercises)+Virtual Therapeutic Garden (20 min of intense visual, auditory, and kinesthetic stimuli through immersion in a garden with the therapist’s voice guiding the patient)	General fitness training (20 min)+relaxation session (10 min) and psychoeducation (10 min)	Intervention: baseline=mean 13.10 (SD 4.26); after treatment=mean 7.33 (SD 3.88); Cohen *d*=1.86, 95% CI 1.26 to 2.45; control: baseline=mean 13.27 (SD 3.80); after treatment=mean 11.57 (SD 5.49); Cohen *d*=0.42, 95% CI 0.04 to 0.79; test used: ANCOVA^c^ (between groups); *P*<.001
Czech et al [35], 2022	Unclear	Poland	Parallel group 2-arm randomized controlled trial+2 time point measures (before and after the intervention)	Sex: female (16/16, 100%); age (y): intervention=mean 50.6 (SD 12.6); control=mean 59.6 (SD 7.9); other: participants with breast cancer	Depressive symptoms measured by Beck Depression Inventory	16 (randomized; intervention: 9; control: 7)	Virtual Therapeutic Garden: intense visual, auditory, and kinesthetic stimuli through garden immersion with the therapist’s voice guiding the patient	Standard of care (not specified)	Intervention: baseline=mean 13.33 (SD 5.57); after treatment=mean 8.11 (SD 6.17); control: baseline=mean 9.00 (SD 7.07); after treatment=mean 7.00 (SD 5.51); test used: 1-way repeated measures ANOVA (for time and groups); *P*=.04
Farahimanesh et al [36], 2023	2021	Iran	Parallel group 2-arm randomized controlled trial+2 time point measures (before and after the intervention)	Sex: intervention=female 18/30 (60%); control=female 15/30 (50%); age (y): intervention=mean 49.1 (SD 10.9); control=mean 49.7 (SD 10.4); other: at least 2 months of social distancing measures related to the COVID-19 pandemic	Depressive symptoms measured by the Depression Anxiety Stress Scale-21	60 (randomized; intervention: 30; control: 30)	COVID Feel Good: a daily intervention with 7 thematic modules, each with two integrated parts: (1) watching a 10-min 360-degree VR video titled Secret Garden+listening to a relaxation induction narrative and (2) social tasks with a different purpose for each day of the wk	No treatment	Intervention: baseline=mean 6.6 (SD 3.1); group 1 after intervention=mean 6 (SD 2.86); group 2 after intervention=mean 5.63 (SD 2.95); control: baseline=mean 6.93 (SD 2.78); group 1 after treatment=mean 6.93 (SD 2.38); group 2 after treatment=mean 6.90 (SD 2.34); test used: ANOVA (for time and groups); *P*=.002
Jóźwik et al [38], 2021	Unclear	Poland	Parallel group 2-arm randomized controlled trial+2 time point measures (before and after the intervention)	Sex: female (26/26, 100%); age (y): mean 65.4 (SD 8.0); intervention=mean 65.6 (SD 10.1); control=mean 65.2 (SD 6.5); other: participants with ischemic heart disease	Depressive symptoms measured by the HADS^d^	52 (randomized; intervention: initial=26, at conclusion=17; control: 26)	Interval training on a cycle ergometer (40 min)+general fitness exercises (40 min)+Virtual Therapeutic Garden (20 min of intense visual, auditory, and kinesthetic stimuli through immersion in a garden with the therapist’s voice guiding the patient)	Interval training on a cycle ergometer (40 min)+general fitness exercises (40 min)+Schultz autogenic training guided by therapist and CD recording	Intervention: baseline=mean 6.14 (SD 3.77); after treatment=mean 4.86 (SD 3.48); mean difference=−1.29 (95% CI −2.12 to −0.46); control: baseline=mean 6.35 (SD 3.91); after treatment=mean 6.53 (SD 3.86); change=0.18 (95% CI −0.16 to 0.52); test used: 2-way repeated measures ANOVA (for time and groups); *P*=.01
Jóźwik et al [37], 2021	2020	Poland	Parallel group 2-arm randomized controlled trial+masked outcome assessor+2time point measures (before and after the intervention)	Sex: intervention=female 17/28 (61%); control=female 25/49 (51%); age (y): intervention=mean 66 (SD 9.7); control=mean 63.9 (SD 6.9); other: participants with coronary artery disease+cardiac rehabilitation phase II	Depressive symptoms measured by the HADS	100 (randomized; intervention: initial=50, at conclusion=28; control: initial=50; at conclusion=49)	Interval training on a cycle ergometer (40 min)+general fitness exercises (40 min)+Virtual Therapeutic Garden (20 min of intense visual, auditory, and kinesthetic stimuli through immersion in a garden with the therapist’s voice guiding the patient)	Interval training on a cycle ergometer (40 min)+general fitness exercises (40 min)+Schultz autogenic training via CD recording (20 min)	Intervention: baseline=mean 6.41 (SD 4.21); after treatment=mean 5.06 (SD 3.88); control: baseline=mean 7.35 (SD 3.80); after treatment=mean 7.27 (SD 4.00); test used: *t* test for independent trials; *P*=.07 for posttreatment measurements
Kiper et al [39], 2022	2020	Poland	Parallel group 2-arm randomized controlled trial+2 time point measures (before and after the intervention)	Sex: intervention=female 17/30 (57%); control=female 13/30 (43%); age (y): intervention=mean 65.5 (SD 6.7); control=mean 65.6 (SD 4.9); other: participants with a history of ischemic stroke and older adult depression diagnosed using the GDS-30 (>10)	Older adult depression measured using the GDS-30; depressive symptoms measured using the HADS (depression subscale)	60 (randomized; intervention: initial=30, at conclusion=22; control: initial=30, at conclusion=17)	First 3 wks: functional rehabilitation (60 min)+Virtual Therapeutic Garden (20 min of intense visual, auditory, and kinesthetic stimuli through immersion in a garden with the therapist’s voice guiding the patient); next 3 weeks: functional rehabilitation (60 min)	First 3 wks: functional rehabilitation (60 min)+Schultz autogenic training via CD recording (20 min); next 3 wks: functional rehabilitation (60 min)	GDS-30—intervention: baseline vs group 1 after treatment=mean difference −6.33 (95% CI −4.42 to −8.24); baseline vs group 2 after treatment=mean difference −6.60 (95% CI −4.69 to −8.51); control: baseline vs group 1 after treatment=mean difference −3.40 (95% CI −1.49 to −5.31); baseline vs group 2 after treatment=mean difference −3.17 (95% CI −1.26 to −5.08); test used: 2-way repeated measures ANOVA (for time and groups); *P*<.01; HADS (depression scale)—intervention: baseline vs group 1 after treatment=mean difference –1.50 (95% CI –0.32 to –3.32); baseline vs group 2 after treatment=mean difference –2.05 (95% CI –0.23 to –3.87); control: baseline vs group 1 after treatment=mean difference 0.79 (95% CI –1.08 to –2.66); baseline vs group 2 after treatment=mean difference 0.73 (95% CI –1.13 to –2.61); test used: 2-way repeated measures ANOVA (for time and groups); *P*<.31
Lakhani et al [40], 2020	2019	Australia	Crossover group 2-arm randomized controlled trial+3 time point measures (1 before and 2 after the intervention)	Sex: group 1=female 0/10 (0%); group 2=female 8/14 (57%); age (y): group 1=mean 56.2 (SD 20.7); group 2=mean 48.0 (SD 16.2); other: participants with spinal cord injury	Depressive symptoms measured using the Patient Health Questionnaire-8	24 (randomized; group 1: initial=10, at conclusion=6; group 2: initial=14, at conclusion=10)	Standard of care (tailored regular rehabilitation involving occupational therapy and physiotherapy)+VR session of environment exposure using the National Geographic app and the YouTube VR app	Standard of care (tailored regular rehabilitation involving occupational therapy and physiotherapy)	Group 1: baseline=mean 5.83 (SD 4.71); group after intervention treatment=mean 3.33 (SD 3.44); control group after treatment=mean 4.17 (SD 5.04); group 2: baseline=mean 5.50 (SD 2.99); control group after treatment=mean 6.30 (SD 1.95); intervention group after treatment=mean 5.90 (SD 3.28); test used: paired *t* test (between groups); *P*=.04 (between baseline and after the control treatment) and *P*=.47 (between after the control treatment and after the intervention treatment); test used: 1-way ANOVA (within groups); *P*=.09 (group 1) and *P*=.83 (group 2)
Paul et al [41], 2022	2020-2021	United States	Parallel group 3-arm randomized controlled trial+4 time point measures (1 before and 3 after the intervention)	Sex: initial=female 7/13 (54%); complete study=female 4/10 (10%); age (y): initial=mean 35.4 (SD 12.3); complete study=mean 34.6 (SD 11.50); other: participants with major depressive disorder diagnosed using the Patient Health Questionnaire-8 (score>10)	Depressive symptoms measured using the Patient Health Questionnaire-9	13 (randomized; intervention: initial=5, at conclusion=3; control 1: 4 and control 2: 4)	Behavioral activation therapy via teleconference platform+VR activities (360-degree YouTube VR videos) after teleconference session (during the wk)	Control 1: verbally asked questions for depression screening using the Patient Health Questionnaire-9 via telephone call; control 2: behavioral activation therapy via teleconference platform+in-person activities after teleconference session (during the wk)	Intervention: baseline vs posttreatment 3=mean difference −5.67; control 1: baseline vs posttreatment 3=mean difference −3.00; control 2: baseline vs posttreatment 3=mean difference −0.25 (no statistical test was used)
Rodrigues et al [42], 2022	2020-2021	Brazil	Parallel group 2-arm randomized controlled trial+2 time point measures (before and after the intervention)	Sex: intervention=female 11/22 (50%); control=female 11/22 (50%); age (y): intervention=mean 48.9 (SD 13.9); control=mean 48.5 (SD 16.9); other: participants with COVID-19 infection	Depressive symptoms measured by the HADS	44 (randomized; intervention: 22; control: 22)	Usual therapy (activities to guide the hospitalization process, coping with the hospitalization process, energy conservation, daily living activity training, cognitive rehabilitation, internet-based call or visit, positioning, mobility joint, functional mobility, kinesiotherapy, assistive technology, sensory stimulation tailored to patient needs)+VR therapy (360-degree videos with images of landscapes and mindfulness techniques)	Usual therapy (activities to guide the hospitalization process, coping with the hospitalization process, energy conservation, daily living activity training, cognitive rehabilitation, internet-based call or visit, positioning, mobility joint, functional mobility, kinesiotherapy, assistive technology, sensory stimulation tailored to patient needs)+VR control (videos with advertisements not related to relaxation and well-being content)	Intervention: baseline=mean 9.83 (SD 4.31); after treatment=mean 7.17 (2.79); Cohen *d*=0.73; control: baseline=mean 15.00 (SD 10.31); after treatment=mean 13.00 (SD 9.49); Cohen *d*=0.20; test used: Wilcoxon test (within groups); *P*=.04 (intervention) and *P*=.08 (control); test used: Mann-Whitney *U* test (between groups); *P*>.05
Rutkowski et al [43], 2021	2020	Poland	Parallel group 2-arm randomized controlled trial+2 time point measures (before and after the intervention)	Sex: intervention=female 21/25 (84%); control=female 20/25 (80%); age (y): intervention=mean 64.4 (SD 5.7); control=mean 67.6 (SD 9.4); other: participants with chronic obstructive pulmonary disease and depression or anxiety diagnosed using the Hamilton Anxiety and Depression Scale (score>8)	Depressive symptoms measured using the HADS	50 (randomized; intervention: 25; control: 25)	Traditional pulmonary rehabilitation program (15-30 min)+Virtual Therapeutic Garden (20 min of intense visual, auditory, and kinesthetic stimuli through immersion in a garden with the therapist’s voice guiding the patient)	Traditional pulmonary rehabilitation program (15-30 min)+Schultz autogenic training session (20 min)	Intervention: baseline=mean 7.96 (SD 2.76); after treatment=mean 6.04 (SD 3.21); control: baseline=mean 6.64 (SD 2.80); after treatment=mean 7.08 (SD 3.56); test used: repeated measures ANOVA (within groups); *P*=.001 (intervention) and *P*=.45 (control)
Rutkowski et al [44], 2022	2021	Poland	Parallel group 2-arm randomized controlled trial+2 time point measures (before and after the intervention)	Sex: female 20/32 (69%); age (y): mean 57.8 (SD 4.9); other: participants with COVID-19 infection	Depressive symptoms measured using the HADS	32 (randomized; intervention: 16; control: 16)	Pulmonary rehabilitation program+VR-based exercise capacity training: bicycle ergometer+Virtual Park VR experience (bicycle trip in an island simulation synced with ergometer)+VR-based relaxation (Virtual Therapeutic Garden: intense visual, auditory, and kinesthetic stimuli through immersion in a garden with the therapist’s voice guiding the patient)	Pulmonary rehabilitation program+traditional exercise capacity training: bicycle ergometer exercise+Schultz autogenic training	Intervention: baseline=mean 6.9 (SD 3.9); after treatment=mean 4.7 (SD 3.5); control: baseline=7.64 (4.5); after treatment=6.6 (4.8); test used: paired *t* test (within groups); *P*=.008 (intervention) and *P*=.02 (control)
Szczepańska-Gieracha et al [45], 2021	Unclear	Poland	Parallel group 2-arm randomized controlled trial+2 time point measures (before and after the intervention)	Sex: female 20/32 (63%); intervention=female 9/15 (60%); control=female 11/17 (65%); age (y): mean 68.9 (SD 6.3); intervention=mean 69.5 (SD 7.5); control=mean 68.4 (SD 5.0); other: participants with coronary artery disease	Depressive symptoms measured using the HADS	34 (randomized; intervention: initial=17, at conclusion=15; control: 17)	Cardiac rehabilitation (1.5 h): cardiological training individually prescribed based on an exertion test and heart rate reserve+Virtual Therapeutic Garden (20 min of intense visual, auditory, and kinesthetic stimuli through immersion in a garden with the therapist’s voice guiding the patient)	Cardiac rehabilitation (1.5 h): cardiological training individually prescribed based on an exertion test and heart rate reserve+Schultz autogenic training (20 min) delivered by a therapist and CD recording	Intervention: baseline=mean 9.00 (SD 2.39); after treatment=mean 6.93 (SD 3.01); Cohen *d*=0.89; *P*=.003; control: baseline=mean 9.24 (SD 2.41); after treatment=mean 9.35 (SD 2.50); Cohen *d*=−0.15; test used: paired *t* test (within groups); *P*=.003 (intervention) and *P*=.54 (control)
Szczepańska-Gieracha et al [46], 2021	2019	Poland	Parallel group 2-arm randomized controlled trial+masked outcome assessor+3time point measures (1 before and 2 after the intervention)	Sex: female 23/23 (100%); age (y): mean 70.7 (SD 13.7); intervention=mean 70.2 (SD 4.9); control=mean 71.2 (SD 4.4); other: female older adult with severe depression (GDS-30 score>10) and nonrespondent to treatment program	Older adult depression measured using the GDS-30	25 (randomized; intervention: initial=13, at conclusion=11; control: 12)	Support group meetings: general fitness training (40 min) and relaxation exercises, as well as health-promoting education and psychoeducation (20 min)+Virtual Therapeutic Garden (intense visual, auditory, and kinesthetic stimuli through immersion in a garden with the therapist’s voice guiding the patient)	Support group meetings: general fitness training (40 min) and relaxation exercises, as well as health-promoting education and psychoeducation (20 min)	Intervention: baseline=mean 12.27 (SD 4.45); group 1 after treatment=mean 8.27 (SD 3.60); group 2 after treatment=mean 7.27 (SD 2.57); control: baseline=mean 12.25 (4.53); group 1 after treatment=mean 12.75 (4.82); group 2 after treatment=mean 11.83 (2.62); test used: repeated measures ANOVA (within groups); *P*<.001 (intervention) and *P*=.61 (control); test used: repeated measures ANOVA (for time and groups); *P*<.001
Vlake et al [47], 2022	2020-2021	Netherlands	Parallel group 2-arm randomized controlled trial, open label+3 time point measures (1 before and 2 after the intervention)	Sex: intervention=female 10/45 (22%); control=female 16/44 (64%); age (y): intervention=median 61 (IQR 54-65); control=median 59 (IQR 51-65); other: participants included survivors of COVID-19 infection who had been discharged from the ICU^e^	Depressive symptoms measured using the HADS	89 (randomized; intervention: initial=45; 1-month assessment=45 and 3-month assessment=39; control: initial=44; 1-month assessment=44 and 3-month assessment=38)	Intensivist consultation (60 min): revision of treatment, screening for postintensive care syndrome–related impairment, and referral to appropriate health provider+intensive care unit VR (14 min of an informational VR video aimed to immerse patients in the ICU environment and provide voice-over explanations regarding different facets of the ICU and ICU treatment, which consisted of 6 scenes covering topics such as ICU equipment, procedures, and COVID-19)	Intensivist consultation (60 min): treatment revision, postintensive care syndrome–related impairment screening, and referral to the appropriate health provider	Intervention: baseline=18%; 1 month after treatment=24%; 3 months after treatment=23%; control: baseline=33%; 1 month after treatment=41%; 3 months after treatment=29%; test used=logistic regression (for time points); *P*=.57 (baseline vs first time point 1 month after the treatment) and *P*=.51 (baseline vs second time point 3 months after the treatment)
Zhang et al [48], 2022	2021	China	Parallel group 2-arm randomized controlled trial+2 time point measures (before and after the intervention)	Sex: intervention=female 38/38 (100%); control=female 39/39 (100%); age (y): intervention=mean 52.3 (SD 7.7); control=mean 51.0 (SD 7.9); other: participants with a history of breast cancer and 2 courses of chemotherapy completed	Depressive symptoms measured using the Self-Rating Depression Scale	90 (randomized; intervention: initial=45, at conclusion=38; control: initial=45, at conclusion=39)	Care as usual+VR-CALM^f^ intervention (30 min): immersion in calming and beautiful virtual environments (such as the seaside or Butterfly Valley) while receiving CALM therapy delivered by a trained therapist	Care as usual	Intervention: baseline=mean 51.320 (SD 11.552); after treatment=mean 46.630 (SD 9.824); control: baseline=mean 48.640 (SD 4.934); after treatment=mean 50.210 (SD 3.806); test used: paired *t* test (within groups); *P*≤.001 (intervention) and *P*=.14 (control)
Zhang et al [49], 2023	2021-2022	China	Parallel group 2-arm randomized controlled trial+2 time point measures (before and after the intervention)	Sex: intervention=female 17/30 (57%); control=female 16/30 (53%); age (y): intervention: mean 33.5 (SD 11.1); control: mean 35.3 (SD 10.6); other: participants with leukemia undergoing chemotherapy	Depressive symptoms measured using the Center for Epidemiological Studies Depression Scale	63 (randomized; intervention: initial=32, at conclusion=30; control: initial=31, at conclusion=30)	Usual care: addressing patients’ physiological needs and routine psychological care+VR meditation (20 min of 360-degree videos composed of images of landscapes [beach or forest], background music, and meditation guidance)	Usual care: addressing patients’ physiological needs and routine psychological care	Intervention: baseline=mean 14.23 (SD 8.11); after treatment=mean 11.13 (SD 6.01); control: baseline=mean 14.03 (SD 7.68); after treatment=mean 14.10 (SD 7.18); test used: paired *t* test (within groups); *P*<.001 (intervention) and *P*=.93 (control); test used: independent 2-tailed *t* test (between groups); *P*=.19 (after treatment)

^a^VR: virtual reality.

^b^GDS-30: Geriatric Depression Scale-30.

^c^ANCOVA: analysis of covariance.

^d^HADS: Hospital Anxiety and Depression Scale.

^e^ICU: intensive care unit.

^f^VR-CALM: Managing Cancer and Living Meaningfully based on VR.

Participant demographics showed that, of the 16 reports, 5 (31%) exclusively involved female participants [34,35,37,46,48]; furthermore, 7 (44%) included participants whose mean or median age was >60 years [34,37-39,43,45,46], while only 2 (13%) included participants whose mean age was <45 years [41,49]. Comorbid conditions were noted as inclusion criteria in 15 (94%) of the 16 studies. Among these 15 studies, cancer [35,48,49], heart disease [37,38,45], and COVID-19 infection [42,44,47] were reported in 3 (20%) studies each. Other conditions such as chronic obstructive pulmonary disease [43], social isolation [36], stroke [39], and spinal cord injury [40] were also mentioned.

Of the 16 reviewed reports, 14 (88%) assessed depressive symptoms [34-38,40-45,47-49]. The most widely used instrument was the Hospital Anxiety and Depression Scale (8/16, 50%) [37-39,42-45,47]. Other questionnaires used to measure depressive conditions included the Beck Depression Inventory [35], the Patient Health Questionnaire-8 [40], the Patient Health Questionnaire-9 [41], the Center for Epidemiological Studies Depression Scale [49], the Depression Anxiety Stress Scale-21 [36], and the Self-Rating Depression Scale [48]. In addition to depressive symptoms, older adult depression was explicitly evaluated in 3 (19%) of the studies [34,39,46], using the Geriatric Depression Scale-30.

Regarding the effects of IMTs on depression, only 1 (6%) of the 16 studies reported no improvement in depression scales [47]. Among the 15 studies that demonstrated a positive effect, 11 (73%) reported statistically significant results [34-37,39,43-46,48,49]; 1 (7%) reported nonsignificant results [38]; 2 (13%) reported mixed results [40,42], indicating variability in statistical significance across different measures or outcomes; and 1 (7%) did not perform any statistical analysis [41].

### Technical Aspects of IMT Interventions

The reports included in this review all feature VR interventions, showcasing a broad spectrum of technical characteristics (Table 2). The VR interventions varied, with a majority of the studies (9/16, 56%) implementing the *Virtual Therapeutic Garden* intervention [34,35,37-39,43-46] and 13% (2/16) conducting VR exposure sessions using content from the National Geographic app and the YouTube VR app [40,41]. Unique interventions included *COVID Feel Good* [36], *ICU-VR* [47], *VR-CALM* [48], general VR therapy [42], and individually tailored VR meditation programs.

**Table 2:** Technical characteristics of the immersive technology interventions.

Reports	VR^a^ intervention	Device characteristics	Amount of time used and frequency of use	Intervention duration	Setting of use	Usability assessment	VR design framework used	Degree of guidance
Cieślik et al [34], 2023	Virtual Therapeutic Garden	Type: headset+controllers; specific type: VR TierOne device (VR HTC Vive goggles+HTC Vive controllers)	20 min/session, twice a wk	4 wk	Unclear	Not mentioned	Not mentioned	Self-administered therapy
Czech et al [35], 2022	Virtual Therapeutic Garden	Type: headset+controllers; specific type: VR TierOne device	15 min/session, daily	2 wk (8 sessions)	Hospital (it is not clear whether participants were ambulatory or inpatients)	Not mentioned	Not mentioned	Unclear
Farahimanesh et al [36], 2023	COVID Feel Good	Type: unclear; the study mentions that a head-mounted display or cardboard headset could be used, but there is no information about the VR device type used; specific type: unclear	20 min/session, daily	7 days	Unclear	Not mentioned	Not mentioned	Unclear
Jóźwik et al [37], 2021	Virtual Therapeutic Garden	Type: headset+controllers; specific type: VR TierOne device	Unclear time per session and frequency	Unclear	Hospital (it is not clear whether participants were ambulatory or inpatients)	Not mentioned	Not mentioned	Unclear
Jóźwik et al [38], 2021	Virtual Therapeutic Garden	Type: headset+controllers; specific type: VR TierOne device (HTC Vive PRO VR goggles+unspecified controllers)	20 min/session, 3 times a wk	3 wk (8 sessions)	Hospital (ambulatory)	Not mentioned	Not mentioned	Unclear
Kiper et al [39], 2022	Virtual Therapeutic Garden	Type: headset+controllers; specific type: VR TierOne device (HTC Vive PRO VR goggles+unspecified controllers)	20 min/session; frequency unclear	3 wk (10 sessions)	Hospital (inpatient)	Not mentioned	Methodology of VR clinical trials in health care by an international working group	Unclear
Lakhani et al [40], 2020	VR session of environment exposure using the National Geographic App and the YouTube VR app	Type: headset; specific type: Oculus Go	20 min/session, daily	1 wk (3 sessions)	Hospital (ambulatory)	Not mentioned	Not mentioned	Unclear
Paul et al [41], 2022	VR session of environment exposure using YouTube VR videos	Type: headset; specific type: Limbix	Time per session and frequency unclear	3 wk	Home based	Not mentioned	Not mentioned	Self-administered therapy
Rodrigues et al [42], 2022	VR therapy (360-degree videos with images of landscapes and mindfulness techniques)	Type: smartphone VR headset; specific type: Oculos Realidade Virtual 3D Gamer Warrior JS080 (adaptable with smartphone)	10 min/session, once	1 session	Hospital (inpatient)	Not mentioned	Not mentioned	Predominantly self-help
Rutkowski et al [43], 2021	Virtual Therapeutic Garden	Type: headset+controllers; specific type: VR TierOne device	20 min/session, 5 times a wk	2 wk	Hospital (it is not clear whether participants were ambulatory or inpatients)	Not mentioned	Not mentioned	Unclear
Rutkowski et al [44], 2022	Virtual Therapeutic Garden	Type: headset; specific type: VR TierOne device (VR goggles+controllers)	Unclear time per session, 5 times a wk	3 wk	Hospital (inpatient)	Not mentioned	Not mentioned	Unclear
Szczepańska-Gieracha et al [45], 2021	Virtual Therapeutic Garden	Type: headset; specific type: VR TierOne device (HTC Vive PRO VR goggles+unspecified controllers)	20 min/session, twice a wk	4 wk (8 sessions)	Hospital (inpatient)	Not mentioned	Methodology of VR clinical trials in health care by an international working group	Predominantly self-help
Szczepańska-Gieracha et al [46], 2021	Virtual Therapeutic Garden	Type: headset+controllers; specific type: VR TierOne device	20 min/session, twice a wk	4 wk	Unclear	Not mentioned	Methodology of VR clinical trials in health care by an international working group	Unclear
Vlake et al [47], 2022	ICU-VR	Type: headset+headphones; specific type: Oculus Go+unspecified headphones	14 min/session, once	1 session	Hospital (ambulatory)	Not mentioned	Not mentioned	Predominantly self-help
Zhang et al [48], 2022	VR-CALM	Type: headset+controllers; specific type: unclear	30 min/session; frequency unclear	3 mo (6 sessions)	Hospital (inpatient)	Not mentioned	Not mentioned	Minimal-contact therapy
Zhang et al [49], 2023	Tailored VR meditation	Type: headset; specific type: PRO 6 DOF (Beijing Iqiyi Intelligent Technology)	20 min/session, daily	14 d	Hospital (inpatient)	Not mentioned	Not mentioned	Predominantly self-help

^a^VR: virtual reality.

The immersion devices predominantly used were stand-alone VR headsets (14/16, 88%) [34,35,37-41,43-49]. Of the remaining 2 studies, 1 (50%) used VR headset adapters for smartphones [42], whereas 1 (50%) did not specify the type of device used [36]. The types of specific VR devices varied, with the VR TierOne device (9/16, 56%) [34,35,37-39,43-46] and Oculus Go (2/16, 13%) being the most commonly reported [40,47].

The duration of VR interventions ranged from a single session to daily sessions over up to 8 weeks. However, most of the interventions (10/16, 63%) were administered over a period of 1 day [42,47] to 3 months [48], with 3 weeks (4/16, 25%) being the most common intervention period [38,39,41,44]. The frequency of sessions varied, with daily sessions reported in 4 (25%) of the 16 reports [35,36,40,49] and less frequent sessions noted in the others (12/16, 75%). The majority of the interventions (9/16, 56%) were to be used for 20 minutes per session [34,36,38-40,43,45,46,49]; 3 (19%) of the 16 reports did not specify the session length [37,41,44].

The settings for the interventions were predominantly hospital based (11/16, 69%), with a mix of inpatient, ambulatory, or unclear settings. Only 2 (13%) of the 16 reports specified the intervention as self-administered therapy, with the remaining studies (14/16, 88%) not clearly reporting the degree of guidance provided. None of the reports mentioned usability evaluation as part of their methods or results.

Regarding the VR design framework, only 2 (13%) of the 16 reports mentioned using the *methodology of VR clinical trials in health care by an international working group*. The rest of the reports (14/16, 88%) did not mention any design framework used.

### Therapeutic Approaches

The analysis of therapeutic approaches used in the IMT interventions reveals a varied landscape of techniques (Table 3). Ericksonian psychotherapy was used in 9 (56%) of the 16 reports, indicating its recognized role in VR-based interventions. Mindfulness-based cognitive therapy was the second leading therapeutic approach featured, used in 13% (2/16) of the reports, while cognitive behavioral therapy and behavioral activation were mentioned in only 6% (1/16) of the reports each. Other therapeutic methods included personal psychotherapy based on Managing Cancer and Living Meaningfully (1/16, 6%) [48] and the Roy Adaptation Model (1/16, 6%) [49].

**Table 3:** Therapeutic approaches used in the immersive technology interventions.

Reports	Ericksonian psychotherapy	Mindfulness-based cognitive therapy	Cognitive behavioral therapy	Behavioral activation	Others
Cieślik et al [34], 2023	✓				
Czech et al [35], 2022	✓				
Farahimanesh et al [36], 2023			✓		
Jóźwik et al [37], 2021	✓				
Jóźwik et al [38], 2021	✓				
Kiper et al [39], 2022	✓				
Lakhani et al [40], 2020					
Paul et al [41], 2022				✓	
Rodrigues et al [42], 2022		✓^a^			
Rutkowski et al [43], 2021	✓				
Rutkowski et al [44], 2022	✓				
Szczepańska-Gieracha et al [45], 2021	✓				
Szczepańska-Gieracha et al [46], 2021	✓	✓			
Vlake et al [47], 2022					
Zhang et al [48], 2022					✓
Zhang et al [49], 2023					✓

^a^It is unclear whether all patients in the virtual reality group received a mindfulness-based intervention or just 360-degree videos.

### Implementation Characteristics

Regarding the stages of implementation, 2 (13%) of the 16 articles were at a *preliminary* stage (pilot or feasibility study). By contrast, 6 (38%) of the 16 studies had progressed to the *implementation* stage (referring to prior feasibility results); the remaining articles (8/16, 50%) did not clearly delineate their implementation phase. No article reported an implementation framework used, nor were barriers or enablers of implementation identified.

Most of the reports (15/16, 94%) declared no conflicts of interest; an exception was found in 1 (6%) of the 16 articles, where a potential conflict was disclosed due to an author’s corporate affiliation, emphasizing the importance of transparency in research affiliations and possible biases. Funding sources varied, with half of the studies (8/16, 50%) reporting no external funding, suggesting that a significant portion of research in this area is conducted independently of external financial support. Notably, individual studies were supported by grants from prominent institutions such as the National Science Foundation of China as well as national research grants, demonstrating the global investment in health VR intervention research. All included reports reported ethical oversight, ranging from institutional review boards to national ethics committees.

## Discussion

### Principal Findings

Our scoping review, conducted in accordance with the framework formulated by Arksey and O’Malley [26], identified a total of 16 peer-reviewed articles focusing on the use of IMTs in treating depression. In comparison, other reviews using similar methodologies and research questions have reported varying numbers of studies; for instance, Fodor et al [23] found 24 studies examining the effects of VR interventions on depressive outcomes, whereas Zeng et al [24] included only 5 studies in their review. The larger number of studies in the first review can be attributed to the inclusion of several articles that measured depression as a secondary outcome, which is a common approach in the literature on VR interventions. By contrast, our scoping review strictly included reports that explicitly measured depression as a primary outcome. This was done to specifically target papers that focused on designing and developing interventions addressing this mental disorder. Regarding the second review, the limited number of studies included can be primarily attributed to the authors’ focus on exercise interventions using VR. However, this explanation might not be complete. During our full-text review phase, we identified numerous studies assessing VR interventions related to exercise among the excluded studies (36/52, 69%). Another possible explanation for the limited number of studies is the year of the review’s development: 2018. Bibliometric studies on VR in health care [50] and specifically in depression [51] have shown a significant upward trend since that year, likely linked to the increased availability of VR technologies [52]. This aligns with our findings, where we observed a clear upward trend over the years.

### Secondary Questions

#### From Which Regions or Countries Does the Evidence Come?

Research on IMT interventions for depression care was predominantly conducted in Europe (10/16, 63%), with Poland contributing the most studies (9/16, 56%), indicating a robust regional focus within the field. Our results align with what has been previously observed in the literature, where it is noted that most articles published on VR in depression originate from Europe [51]. This trend denotes a divergence in the topics related to VR in health because most articles on VR in health, in general, have been published in the United States [50].

These figures suggest that European high-income countries exhibit a more consistent and robust research trajectory related to IMT-based treatments for depression. By contrast, other high-income countries such as the United States and Australia and upper–middle-income countries such as Brazil show sporadic participation. Nevertheless, it is essential to approach these figures cautiously, recognizing that the frequency of publications within specific years might not accurately reflect ongoing research interest or the immediacy of research outputs, given the cyclical nature of research funding, project development, and publication processes.

#### Which Technical Aspects of IMTs Have Been Reported in the Evidence?

In our review, no study reported using AR; instead, stand-alone VR headsets emerged as the primary technology, underscoring a trend toward self-contained IMT devices in treating depression. This observation aligns with existing evidence; for example, a systematic review concentrating on the mental health applications of AR did not reveal any applications of this particular IMT modality in either the treatment or the assessment of depression [53].

In terms of duration, most of the VR interventions in our review (10/16, 63%) ranged from 2 to 8 weeks, encompassing 1 to 10 sessions. This aligns with the range of 1 to 16 sessions reported in similar VR studies [23]. In addition, we observed that the predominant setting for these interventions was hospital based (11/16, 69%), with only 6% (1/16) being delivered in home settings. This finding aligns with existing evidence in mindfulness VR interventions, where only 1 of 15 studies was home based [54]. Such a trend indicates a current focus on clinical settings for VR intervention delivery, suggesting potential areas for expansion into more accessible home-based environments.

Only 2 (13%) of the 16 studies reported using a specific IMT design framework, pointing to a potential area for standardization in future research. Conceptual and methodological frameworks are pivotal because they provide a structured approach, align the study’s methodology with its objectives, and facilitate the integration of technology to achieve therapeutic goals [55]. Their application in IMT research is essential for producing reliable and applicable results, particularly in the intricate mental health field [56]. This underuse highlights the need for a more structured and theoretically informed approach in future research, which could enhance the quality, applicability, and standardization of IMT interventions for treating depression.

#### What Therapeutic Approaches Were Used?

Ericksonian psychotherapy was the most common therapeutic approach incorporated into the VR interventions (9/16, 56%). The Ericksonian approach to psychotherapy and hypnosis is based on three key assumptions: (1) the belief in an altered state of consciousness and the existence of specific markers indicating this altered state, (2) the superiority of indirect over direct suggestion in therapy, and (3) the view that a patient’s hypnotizability is a function of the hypnotist’s skill. However, empirical support for the validity of these critical assumptions is limited [57]. Notably, most studies using this approach originated from Poland (9/16, 56%), indicating a geographic concentration of the evidence. Therefore, there is a need to evaluate this intervention in diverse settings to validate its efficacy more broadly. Despite the geographic concentration of Ericksonian therapy within VR interventions, the use of hypnosis and mindfulness techniques can be advantageous in both face-to-face psychotherapy and virtual contexts. These techniques can alleviate life problems and symptoms associated with mental disorders, including depression [58].

Mindfulness-based cognitive therapy has shown effectiveness in reducing depressive symptoms and elucidating the active mechanisms during mindfulness [59,60]. By contrast, cognitive behavioral therapy and behavioral activation are considered therapies with solid evidence for reducing depression [61]. Thus, behavioral therapies may possess a more robust theoretical basis than other treatment models in IMT interventions for depression care, suggesting a potential direction for future research and application.

#### What Are the Barriers and Facilitators to Implementing IMT Interventions for Depression Treatment?

The included studies do not provide evidence on barriers and facilitators to implementation. One possible reason is that we did not include qualitative studies in our scoping review (qualitative research focuses on these types of outcomes). However, a framework for implementing digital mental health interventions identified the key elements: access to the intervention, cost-effectiveness, and user satisfaction, in addition to the evaluation of the effectiveness of the intervention [6].

The primary facilitator for the implementation of IMT interventions for depressive symptoms described in the literature is the availability of evidence supporting the efficacy of the treatment [23,24]. There is also evidence of VR’s acceptability, feasibility, and user satisfaction in mental health settings [62]. However, the cost of VR equipment and the cost of training health professionals may be barriers to access in low- and middle-income countries. In addition, we found no evidence of the cost-effectiveness of IMT interventions on mental health outcomes within the health care system. Therefore, cost-effectiveness and cost-utility studies compared with usual care or other psychological interventions must be developed to provide sufficient evidence to evaluate the implementation of IMT interventions within public health systems.

#### What Outcomes Have Been Evaluated in Studies Examining the Impact of IMT Interventions on Addressing Depression?

In general, all studies used a psychometric scale to assess the impact of the intervention on depressive symptoms, and the instruments used have evidence of reliability and validity; therefore, we considered the results to be adequately assessed. However, there was a high degree of heterogeneity in the instruments used. Some studies used scales focused on hospital settings (eg, the Hospital Anxiety and Depression Scale), others used scales designed for geriatric use (eg, the Geriatric Depression Scale-30), and still others used specialized instruments developed for population use (eg, the Patient Health Questionnaire). Although all instruments assess depressive symptoms, they may assess different forms of the presence of depressive symptoms. Older adults should be considered to have manifestations of depression that are clinically different from those of adults with depression [63]. Therefore, it is essential to consider the setting in which each study was conducted when comparing results.

### Strengths and Limitations

To our knowledge, this study is one of the first to comprehensively identify the current state of research regarding the use of IMT interventions specifically focused on depression. Our work fills essential gaps in existing literature by mapping the current evidence and providing insightful recommendations for future research development. Additional strengths of this study include providing valuable insights into the geographic distribution of research efforts and the range of therapeutic approaches used. This contributes significantly to a deeper understanding of the field and highlights areas where further research is needed.

This study has significant limitations that deserve consideration. First, our selection process, involving screening by title and abstract followed by full-text review, may have introduced selection bias if relevant studies were inadvertently excluded due to inadequate information in titles or abstracts. To mitigate this risk, we used a thorough screening process with multiple reviewers for each study, aiming to reduce selection bias.

Second, our search was limited to articles published in English, potentially leading to language bias by excluding relevant studies in other languages. While future reviews could include studies in multiple languages for a broader range of evidence, this limitation did not significantly narrow the scope of our review because a substantial portion of the evidence in this field is published in English.

Third, the studies included were restricted to RCTs. While RCTs are considered the gold standard in clinical research because they provide high-quality evidence on the efficacy of interventions, this restriction may have limited the comprehensiveness of our review. Specifically, valuable exploratory, observational, and qualitative studies that could provide insights into the implementation, user experience, and broader contextual factors related to IMTs in depression care were excluded. Future reviews could consider including a wider range of study designs to provide a more holistic view of the field, thereby enhancing our understanding of the efficacy of these technologies and their practical application, barriers to implementation, and patient perspectives.

Fourth, we did not consider the high cost of IMT equipment, the training of health professionals, and other economic aspects in the study extraction process, which could be significant barriers, especially in low- and middle-income countries. This oversight underscores the necessity for cost-effectiveness and cost-utility studies to assess the feasibility of IMT interventions in diverse health care settings.

Fifth, the considerable variability in the psychometric scales used across the studies could impact result comparability. We recognize this heterogeneity and recommend that future research consider setting and population-specific scales to improve comparability.

Finally, our scoping review did not include a formal quality appraisal of the included studies. However, it is important to note that our review focused exclusively on RCTs published in peer-reviewed journals. This focus on RCTs, combined with the peer-review process, increased the likelihood that high-quality studies were included. While this approach does not guarantee the quality of each study, it does suggest that the evidence base we have mapped is likely to be more rigorous and high-quality research compared to broader inclusion criteria.

### Conclusions

Our scoping review on the use of IMTs for treating depression identified 16 peer-reviewed articles predominantly focused on stand-alone VR headsets. Most of the research was concentrated in Europe (10/16, 63%), specifically Poland (9/16, 56%), suggesting a need for more geographically diverse studies. Furthermore, the therapeutic approaches in these studies largely centered around Ericksonian psychotherapy; however, given the limited empirical support for the fundamental assumptions of Ericksonian psychotherapy and the geographic bias, there is a clear need for exploring a variety of therapeutic approaches in IMT interventions for depression care.

A notable gap in the literature is the absence of AR approaches for depression treatment in the studies reviewed. This points toward an opportunity for future research in this area. In addition, while VR shows promise in mental health settings, concerns about the cost and accessibility, particularly in low- and middle-income countries, highlight the need for more research into the cost-effectiveness of these interventions.

In summary, while the use of IMTs in treating depression shows promise, our review indicates the need for more diverse, inclusive, and comprehensive research. Future studies should address the identified gaps, particularly in AR, cost-effectiveness, and geographic diversity, to fully harness the potential of IMT interventions in depression care.

## Appendix

Multimedia Appendix 1 The PRISMA-ScR (Preferred Reporting Items for Systematic Reviews and Meta-Analyses extension for Scoping Reviews) checklist.

Multimedia Appendix 2 Search strategies by database consulted.

Multimedia Appendix 3 List of articles excluded from the full-text review (36/52, 69%).

## Abbreviations

AR: augmented reality
IMT: immersive technology
PRISMA-ScR: Preferred Reporting Items for Systematic Reviews and Meta-Analyses extension for Scoping Reviews
RCT: randomized clinical trial
VR: virtual reality
