# Supplementary material for: Immersive Technologies for Depression Care: Scoping Review
Source: JMIR Ment Health. 2024 Apr 25;11:e56056. doi: 10.2196/56056 (PMC11082738; doi:10.2196/56056)
Supplement: Multimedia Appendix 2 [file mental_v11i1e56056_app2.docx]

**Multimedia Appendix 1: Seach strategies by database consulted.**

MEDLINE (via Pubmed)

|  | Search strategy | Retrieves |
| --- | --- | --- |
| #1 | "Depression"[Mesh] OR "Depressive Disorder"[Mesh] OR depressive*[tiab] OR depression[tiab] | 537,163 |
| #2 | "Virtual Reality"[Mesh] OR "virtual reality"[tiab] OR "Augmented Reality"[Mesh] OR "augmented reality"[tiab] OR "VR headset"[tiab] OR "VR glasses"[tiab] OR "virtual environment"[tiab] OR "virtual world"[tiab] OR metaverse[tiab] OR meta-verse[tiab] | 24,964 |
|  | "Randomized Controlled Trial"[Publication Type] OR "Randomized Controlled Trials as Topic"[Mesh] OR “randomized clinical trial”[tiab] OR RCT[tiab] OR (randomized[tiab] AND “clinical trial”[tiab]) | 802,406 |
| #3 | #1 AND #2 AND #3 | 119 |

SCOPUS

|  | Search strategy | Retrieves |
| --- | --- | --- |
| #1 | TITLE-ABS-KEY ( depressive OR depression ) | 933,322 |
| #2 | TITLE-ABS-KEY ( "virtual reality" OR "augmented reality" OR "VR headset" OR "VR glasses" OR "virtual environment" OR "virtual world" OR "metaverse" OR "meta-verse" ) | 215,687 |
| #3 | TITLE-ABS-KEY ( “randomized clinical trial” OR RCT) OR TITLE-ABS-KEY ( randomized AND “clinical trial”) | 638,457 |
| #3 | #1 AND #2 AND #3 | 116 |

Web of Sciences

|  | Search strategy | Retrieves |
| --- | --- | --- |
| #1 | (TS=(depressive)) OR TS=(depression) | 696,524 |
| #2 | (((((((TS=(virtual reality)) OR TS=(augmented reality)) OR TS=(VR headset)) OR TS=(VR glasses)) OR TS=(virtual environment)) OR TS=(virtual world)) OR TS=(metaverse)) OR TS=(meta-verse) | 92,867 |
| #3 | (((TS=(randomized )) AND TS=(clinical trial)) OR TS=(randomized clinical trial)) OR TS=(RCT) | 361,876 |
| #3 | #1 AND #2 AND #3 | 116 |

PsycInfo (via EBSCO)

|  | Search strategy | Retrieves |
| --- | --- | --- |
| #1 | TX depressive OR TX depression | 421,262 |
| #2 | TX virtual reality OR TX augmented reality OR TX vr headset OR TX vr glasses OR TX virtual environment OR TX virtual world OR TX metaverse OR TX meta-verse | 18,793 |
| #3 | (TX clinical trial AND TX randomized) OR TX randomized clinical trial OR TX RCT | 44,462 |
| #4 | #1 AND #2 | 66 |

Embase

|  | Search strategy | Retrieves |
| --- | --- | --- |
| #1 | 'depression'/exp OR 'depression assessment'/exp OR 'depression OR 'depressive' | 947,905 |
| #2 | 'virtual reality head mounted display'/exp OR 'virtual reality head mounted display' OR 'virtual reality'/exp OR 'virtual reality' OR 'augmented reality'/exp OR 'augmented reality' OR 'vr headset'/exp OR 'vr headset' OR 'vr glasses'/exp OR 'vr glasses' OR 'virtual environment'/exp OR 'virtual environment' OR 'virtual world' OR 'metaverse'/exp OR 'metaverse' OR 'meta-verse' | 42,111 |
| #3 | 'randomized controlled trial'/exp OR 'randomized controlled trial' OR 'randomized clinical trial' OR 'rct' OR (randomized AND ('clinical trial'/exp OR 'clinical trial')) | 1,179,935 |
| #4 | #1 AND #2 | 366 |

IEEE Xplore

|  | Search strategy | Retrieves |
| --- | --- | --- |
| #1 | ("Full Text & Metadata":depression OR ("Full Text & Metadata":depressive ONEAR/1 ("Full Text & Metadata":symptom OR "Full Text & Metadata":disease OR "Full Text & Metadata":disorder))) | 11,673 |
| #2 | ("Full Text & Metadata": "virtual reality" OR "Full Text & Metadata": "augmented reality" OR "Full Text & Metadata":"VR headset" OR "Full Text & Metadata":"VR glasses" OR "Full Text & Metadata":"virtual environment" OR "Full Text & Metadata":"virtual world" OR "Full Text & Metadata":"metaverse" OR "Full Text & Metadata":"meta-verse") | 21,961 |
|  | (((Full Text & Metadata:clinical trial) AND (Full Text & Metadata:randomized))) OR ((Full Text & Metadata:randomized clinical trial) OR (Full Text & Metadata:rct)) | 3,346 |
| #3 | #1 AND #2 AND #3 | 311 |

Cochrane Library: in Cochrane Reviews, Cochrane Protocols and Trials

|  | Search strategy | Retrieves |
| --- | --- | --- |
| #1 | MeSH descriptor: [Depression] explode all trees OR MeSH descriptor: [Depressive Disorder] explode all trees OR (depression):ti,ab,kw OR (depressive):ti,ab,kw | 104,814 |
| #2 | MeSH descriptor: [Virtual Reality] explode all trees OR MeSH descriptor: [Augmented Reality] explode all trees OR ("virtual reality"):ti,ab,kw OR ("augmented reality"):ti,ab,kw OR ("VR headset"):ti,ab,kw OR ("VR glasses"):ti,ab,kw OR ("virtual environment"):ti,ab,kw OR ("virtual world"):ti,ab,kw OR ("metaverse "):ti,ab,kw OR ("meta-verse"):ti,ab,kw | 6,702 |
|  | MeSH descriptor: [Randomized Controlled Trial] explode all trees OR (randomized clinical trial):ti,ab,kw OR (rct):ti,ab,kw OR ((randomized):ti,ab,kw AND (clinical trial):ti,ab,kw) | 654,703 |
| #3 | #1 AND #2 | 231 |
