# Supplementary material for: Immersive Technologies for Depression Care: Scoping Review
Source: JMIR Ment Health. 2024 Apr 25;11:e56056. doi: 10.2196/56056 (PMC11082738; doi:10.2196/56056)
Supplement: Multimedia Appendix 3 [file mental_v11i1e56056_app3.docx]

**Multimedia Appendix 2.** List of articles excluded in the full-text review (n=36).

| Title | Year | Authors | Exclusion reasons |
| --- | --- | --- | --- |
| (PO-128) Remote Virtual Reality Exposure Therapy for Post-Intensive Care Syndrome | 2022 | Jones, T. and Sisti, J. and Sisti, A. and Agarwal, S. and igo, G. and Zabinski, J. | Wrong publication type |
| [Effect of virtual reality video-based pre-discharge psychological intervention on the post-discharge emotions of patients with deep facial burns: a prospective randomized controlled study] | 2021 | He, T. T. and Zhang, X. H. and Kong, X. L. and Cheng, D. and Wu, W. W. | Foreign language |
| 1717P Use of immersive virtual reality for management of anxiety and depression among chemotherapy-naïve Filipino breast cancer outpatients in a national university hospital | 2021 | Ando, M. M. and Mendoza, M. J. and Leones, L. M. and Ting, F. I. and Sacdalan, D. B. | Wrong publication type |
| A multicomponent digital intervention to promote help-seeking for mental health problems and suicide in sexual and gender diverse young adults: A randomized controlled trial | 2023 | Han, M. and Wang, Y. and Zhang, Y. and Wang, Y. and Ou, J. and Ren, D. and Cai, C. and Liu, K. and Li, R. and Han, J. and Chen, R. | Wrong intervention |
| A RCT of a Psychological Health Promotion Program for Women with SCI:Examining Efficacy and Mechanisms | 2022 | Robinson-Whelen, S. and Hughes, R. and Gonzalez, D. and Norah-Davis, L. and Luis, L. N. and Ngan, E. and Taylor, H. | Wrong publication type |
| Adaptive conjunctive cognitive training (ACCT) in virtual reality for chronic stroke patients: A randomized controlled pilot trial | 2020 | Maier, M. and Ballester, B. R. and Leiva Bañuelos, N. and Duarte Oller, E. and Verschure, P. F. M. J. | Wrong outcome |
| An eight-week, web-based mindfulness virtual community intervention for students' mental health: Randomized controlled trial | 2020 | Ahmad, F. and El Morr, C. and Ritvo, P. and Othman, N. and Moineddin, R. and Ashfaq, I. and Bohr, Y. and Ferrari, M. and Fung, W. L. A. and Hartley, L. and Maule, C. and Mawani, A. and McKenzie, K. and Williams, S. | Wrong intervention |
| Can we treat depression with self-compassion using virtual reality? | 2022 | Isrctn | Protocol |
| Development and Evaluation of a Cost-Effective Virtual Reality Tool for Patient Education Before Radiotherapy Treatment | 2021 | Duan, J. | Wrong publication type |
| Effect of virtual reality PTSD treatment on mood and neurocognitive outcomes | 2014 | McLay, R. and Ram, V. and Murphy, J. and Spira, J. and Wood, D. P. and Wiederhold, M. D. and Wiederhold, B. K. and Johnston, S. and Reeves, D. | Wrong outcome |
| Effects of game-based virtual reality on health-related quality of life in chronic stroke patients: A randomized, controlled study | 2015 | Shin, J. H. and Bog Park, S. and Ho Jang, S. | Wrong intervention |
| Effects of Receptive Music Therapy Combined With Virtual Reality on Prevalent Symptoms in Patients With Advanced Cancer | 2022 | Agustina Iturri | Protocol |
| GOODRENAL: A FEASIBILITY STUDY OF AN INTRADIALYTIC HOLISTIC VIRTUAL-REALITY PLATFORM | 2023 | Segura-Ortí, E. and Maria Avesani, C. and Clyne, N. and Garcia-Testal, A. and Kouidi, E. and Lozano-Quilis, J. A. and Mesa-Gresa, P. and Van Craenenbroeck, A. and Cana-Poyatos, A. and Lauer, A. and Lindholm, B. and Marín, A. E. and Michou, V. and Juhlin, A. K. | Wrong publication type |
| Immersive reminiscence therapy in french nursing homes and long-term care units: First comparative results between generic and customized virtual reality scenarios | 2022 | Anne-Julie, V. C. and Cass and ra, Q. and Olivier, G. | Wrong publication type |
| Immersive virtual reality as a method supporting pulmonary rehabilitation: Evaluation of the intensity of depressive and anxiety symptoms and stress levels | 2021 | Rutkowski, S. and Szczegielniak, J. and Szczepańska-Gieracha, J. | Wrong publication type |
| Innovative technology based interventions for psychological treatment of common mental disorders | 2020 | Donker, T. and Kleiboer, A. | Wrong publication type |
| Intensive Care specific Virtual Reality (ICU-VR) improves Post-Intensive Care Syndrome-related psychological sequelae in survivors of critical illness | 2020 | Vlake, J. H. and Wils, E. J. and Van Bommel, J. and Korevaar, T. and Gommers, D. and Van Genderen, M. | Wrong publication type |
| Intensive Care Unit specific Virtual Reality (ICU-VR) to improve psychological impairments in survivors of COVID-19; a multicentre, randomised controlled trial | 2020 | Vlake, J. H., Van Bommel, J., Wils, E. J., Korevaar, T. I., Hellemons, M. E., Schut, A. F., ... & Van Genderen, M. E. | Protocol |
| Intensive Care Unit-specific Virtual Reality for COVID-19 ICU survivors | 2021 | Vlake, J. H. and Van Bommel, J. and Wils, E. J. and Bienvenu, O. J. and Hellemons, M. and Korevaar, T. and Schut, A. and Labout, J. and Van Bavel, M. and Schreuder, L. and Gommers, D. and Van Genderen, M. | Wrong publication type |
| Investigating the effects of a virtual reality-based stress management programme on inpatients with mental disorders: A pilot randomised controlled trial | 2021 | Tan, H. L. E. and Chng, C. M. L. and Lau, Y. and Klainin-Yobas, P. | Wrong outcome |
| Mindfulness and virtual reality in adults with adhd: Results of a randomised, controlled clinical trial | 2020 | Ramos-Quiroga, J. A. | Wrong publication type |
| Mindfulness in University Students. ATENEU Program | 2023 | ALICIA SANCHEZ PEREZ et al. | Protocol |
| One-year randomized trial comparing virtual reality-assisted therapy to cognitive-behavioral therapy for patients with treatment-resistant schizophrenia | 2021 | Dellazizzo, L. and Potvin, S. and Phraxayavong, K. and Dumais, A. | Wrong outcome |
| Pilot randomised controlled trial of Help4Mood, an embodied virtual agent-based system to support treatment of depression | 2016 | Burton, Christopher and Tatar, Aurora Szentagotai and McKinstry, Brian and Matheson, Colin and Matu, Silviu and Moldovan, Ramona and Macnab, Michele and Farrow, Elaine and David, Daniel and Pagliari, Claudia and Blanco, Antoni Serrano and Wolters, Maria | Wrong intervention |
| Prehabilitative Virtual Reality Mindfulness and Personalized Physical Activity for Hemodialysis Patients With Depressive Symptoms: A Feasibility Study | 2022 | Burrows, B. and King, A. and Morgan, A. and Wilund, K. R. | Protocol |
| Recovery after prolonged ICU treatment in patients with COVID-19 | 2021 | Parotto, M. and Myatra, S. N. and Munblit, D. and Elhazmi, A. and Ranzani, O. T. and Herridge, M. S. | Wrong publication type |
| T29DEVELOPMENT OF THE ASSESSMENT-BASED BIOMARKERS AND THE TAILORED PSYCHOEDUCATION PROGRAM FOR DEPRESSIVE DISORDER USING VIRTUAL REALITY | 2019 | Ryu, J. and Choi, S. W. and Seok, J. H. | Wrong publication type |
| The benefits and acceptability of virtual reality interventions for women with metastatic breast cancer in their homes; a pilot randomised trial | 2022 | Reynolds, L. M. and Cavadino, A. and Chin, S. and Little, Z. and Akroyd, A. and Tennant, G. and Dobson, R. and Broom, R. and Gautier, A. | Wrong outcome |
| The Effect of Maternal–Foetal Attachment–Based Training Programme on Maternal Mental Health Following an Unintended Pregnancy | 2023 | Mahmoudi, P. and Elyasi, F. and Nadi, A. and Ahmad Shirvani, M. | No access is possible |
| Use of immersive virtual reality for management of anxiety and depression among chemotherapy-naïve Filipino breast cancer outpatients in a national university hospital | 2022 | Ando, M. | Wrong publication type |
| Virtual Reality Combined With rTMS for the Treatment of Depression : a Randomized Clinical Trial | 2017 | Nct | Wrong publication type |
| Virtual Reality Reward Training for Anhedonia: A Pilot Study | 2021 | Chen, K. and Barnes-Horowitz, N. and Treanor, M. and Sun, M. and Young, K. S. and Craske, M. G. | Wrong study design |
| Virtual Reality, Mood, and Sedentary Behaviour After Stroke | 2019 | Nct | Protocol |
| Virtual Reality-Reward Training for Anhedonia | 2022 | Nct | Protocol |
| Promoting psychological health in women with SCI: Development of an online self-esteem intervention | 2020 | Robinson-Whelen, S. and Hughes, R. B. and Taylor, H. B. and Markley, R. and Vega, J. C. and Nosek, T. M. and Nosek, M. A. | Wrong intervention |
| Virtual Reality-Based Early Neurocognitive Stimulation in Critically Ill Patients: A Pilot Randomized Clinical Trial | 2021 | Navarra-Ventura, G. and Gomà, G. and Haro, C. and Jodar, M. and Sarlabous, L. and Hern and o, D. and Bailón, R. and Ochagavía, A. and Blanch, L. and López-Aguilar, J. and Fernández-Gonzalo, S. | Wrong intervention |
